# Supplementary material for: The effect of perioperative probiotics and synbiotics on postoperative infections in patients undergoing major liver surgery: a meta-analysis of randomized controlled trials
Source: PeerJ. 2025 Feb 17;13:e18874. doi: 10.7717/peerj.18874 (PMC11841616; doi:10.7717/peerj.18874)
Supplement: Supplemental Information 2 [file peerj-13-18874-s002.docx]

**Supplementary Material 2: Searching strategies**

Pubmed 57

#1 probiotics [Title/Abstract] OR probiotic [Title/Abstract] OR prebiotics [Title/Abstract] OR prebiotic [Title/Abstract] OR symbiotic [Title/Abstract] OR synbiotics [Title/Abstract] OR probiotics [MeSH] OR prebiotics [MeSH] OR synbiotics [MeSH]

#2 hepatectomy [Title/Abstract] OR hepatectomy [MeSH] OR liver resection [Title/Abstract] OR liver transplant* [Title/Abstract] OR hepatic resection [Title/Abstract] OR hepatic transplant* [Title/Abstract] OR abdominal surgery [Title/Abstract] OR hepatobiliary [Title/Abstract] OR liver transplantation [MeSH]

#3 randomised [Title/Abstract] OR randomized [Title/Abstract] OR randomized control trial [Publication Type]

#1 AND #2 AND #3

Embase 75

#1 ‘probiotic*’:ab,ti OR ‘prebiotic*’:ab,ti OR ‘symbiotic*’:ab,ti

#2 ‘hepatectomy’:ab,ti OR ‘liver resection’:ab,ti OR ‘hepatic resection’:ab,ti OR ‘liver transplantation’:ab,ti OR ‘hepatic transplantation’:ab,ti OR ‘abdominal surgery’:ab,ti OR ‘hepatobiliary’:ab,ti

#3 (randomized control trial):ab,ti OR ‘random*’:ab,ti

#1 AND #2 AND #3

Scopus 264

#1 TITLE-ABS-KEY (probiotic*) OR TITLE-ABS-KEY (prebiotic*) OR TITLE-ABS-KEY (symbiotic*)

#2 TITLE-ABS-KEY (hepatectomy) OR TITLE-ABS-KEY (liver resection) OR TITLE-ABS-KEY (hepatic resection) OR TITLE-ABS-KEY (liver transplantation) OR TITLE-ABS-KEY (hepatic transplantation) OR TITLE-ABS-KEY (abdominal surgery) OR TITLE-ABS-KEY (hepatobiliary)

#3 TITLE-ABS-KEY (random*) OR TITLE-ABS-KEY (randomized control trial)

#1 AND #2 AND #3

Cochrane Library 142

#1 ‘probiotic*’:ti,ab,kw OR ‘prebiotic*’:ti,ab,kw OR ‘symbiotic*’: ti,ab,kw

#2 (hepatectomy):ti,ab,kw OR ‘liver resection’:ti,ab,kw OR ‘hepatic resection’:ti,ab,kw OR ‘liver transplantation’:ti,ab,kw OR ‘hepatic transplantation’:ti,ab,kw OR ‘abdominal surgery’:ti,ab,kw OR ‘hepatobiliary’:ti,ab,kw

#3 ‘random*’: ti,ab,kw OR (randomized control trial): ti,ab,kw

#1 AND #2 AND #3
