# Supplementary material for: The effect of perioperative probiotics and synbiotics on postoperative infections in patients undergoing major liver surgery: a meta-analysis of randomized controlled trials
Source: PeerJ. 2025 Feb 17;13:e18874. doi: 10.7717/peerj.18874 (PMC11841616; doi:10.7717/peerj.18874)
Supplement: Supplemental Information 3 [file peerj-13-18874-s003.docx]

**Supplementary Material 3:** List of excluded studies with reasons

| **Author, year and reference** | **Reason for exclusion** |
| --- | --- |
| Wang et al. (2023) [1] | Meta-analysis |
| Xiang et al. (2021) [2] | Meta-analysis |
| Wauters et al. (2021) [3] | Patients without liver surgeries |
| Orłowska et al. (2021) [4] | Patients without liver surgeries |
| Folwarski et al. (2021) [5] | Patients without liver surgeries |
| Wang et al. (2021) [6] | Patients without liver surgeries |
| Ma et al. (2021) [7] | Meta-analysis |
| Grąt et al. (2020) [8] | Non-randomized controlled trial |
| Darbandi et al. (2020) [9] | Review |
| Chowdhury et al. (2020) [10] | Meta-analysis |
| Iida et al. (2020) [11] | Non-randomized controlled trial |
| Gan et al. (2019) [12] | Meta-analysis |
| Franko et al. (2019) [13] | Patients without liver surgeries |
| Yokoyama et al. (2017) [14] | Review |
| Lytvyn et al. (2016) [15] | Meta-analysis |
| Yokoyama et al. (2016) [16] | Patients without liver surgeries |
| van der Beek et al. (2015) [17] | Improper intervention and control methods |
| Sommacal et al. (2015) [18] | Patients without liver surgeries |
| Liu et al. (2015) [19] | Patients without liver surgeries |
| Zhang et al. (2013) [20] | Non-randomized controlled trial |
| Okazaki et al. (2013) [21] | Patients without liver surgeries |
| Jeppsson et al. (2011) [22] | Review |
| Diepenhorst et al. (2011) [23] | Patients without liver surgeries |
| Rifatbegovic et al. (2010) [24] | No concerned outcomes |
| Pitsouni et al. (2009) [25] | Meta-analysis |
| Gurusamy et al. (2008) [26] | Review |
| Rayes et al. (2007) [27] | Patients without liver surgeries |
| Anderson et al. (2004) [28] | Patients without liver surgeries |
| Woodcock et al. (2004) [29] | Improper intervention and control methods |
| McNaught et al. (2002) [30] | Patients without liver surgeries |

**Reference**

1. Wang X, Jin X, Li H, Zhang X, Chen X, Lu K, Chu C: **Effects of various interventions on non-alcoholic fatty liver disease (NAFLD): A systematic review and network meta-analysis**. *Front Pharmacol* 2023, **14**:1180016.

2. Xiang Y, Zhang S, Cui Z, Yang Y: **Exploring the effect of microecological agents on postoperative immune function in patients undergoing liver cancer surgery: a systematic review and meta-analysis**. *Ann Palliat Med* 2021, **10**(11):11615-11627.

3. Wauters L, Slaets H, De Paepe K, Ceulemans M, Wetzels S, Geboers K, Toth J, Thys W, Dybajlo R, Walgraeve D *et al*: **Efficacy and safety of spore-forming probiotics in the treatment of functional dyspepsia: a pilot randomised, double-blind, placebo-controlled trial**. *Lancet Gastroenterol Hepatol* 2021, **6**(10):784-792.

4. Orłowska E, Czubkowski P, Wołochowska K, Jarzębicka D, Motyl I, Socha P: **Assessment of Lactobacillus casei rhamnosus (LGG) therapy in children with biliary atresia - Randomized placebo controlled trial**. *Clin Res Hepatol Gastroenterol* 2021, **45**(6):101753.

5. Folwarski M, Dobosz M, Małgorzewicz S, Skonieczna-Żydecka K, Kaźmierczak-Siedlecka K: **Effects of Lactobacillus rhamnosus GG on early postoperative outcome after pylorus-preserving pancreatoduodenectomy: a randomized trial**. *Eur Rev Med Pharmacol Sci* 2021, **25**(1):397-405.

6. Wang P, Yin X, Chen G, Li L, Le Y, Xie Z, Ouyang W, Tong J: **Perioperative probiotic treatment decreased the incidence of postoperative cognitive impairment in elderly patients following non-cardiac surgery: A randomised double-blind and placebo-controlled trial**. *Clin Nutr* 2021, **40**(1):64-71.

7. Ma M, Wang X, Li J, Jiang W: **Efficacy and safety of probiotics and prebiotics in liver transplantation: A systematic review and meta-analysis**. *Nutr Clin Pract* 2021, **36**(4):808-819.

8. Grąt M, Grąt K, Krawczyk M, Lewandowski Z, Krasnodębski M, Masior Ł, Patkowski W, Zieniewicz K: **Post-hoc analysis of a randomized controlled trial on the impact of pre-transplant use of probiotics on outcomes after liver transplantation**. *Sci Rep* 2020, **10**(1):19944.

9. Darbandi A, Mirshekar M, Shariati A, Moghadam MT, Lohrasbi V, Asadolahi P, Talebi M: **The effects of probiotics on reducing the colorectal cancer surgery complications: A periodic review during 2007-2017**. *Clin Nutr* 2020, **39**(8):2358-2367.

10. Chowdhury AH, Adiamah A, Kushairi A, Varadhan KK, Krznaric Z, Kulkarni AD, Neal KR, Lobo DN: **Perioperative Probiotics or Synbiotics in Adults Undergoing Elective Abdominal Surgery: A Systematic Review and Meta-analysis of Randomized Controlled Trials**. *Ann Surg* 2020, **271**(6):1036-1047.

11. Iida H, Sasaki M, Maehira H, Mori H, Yasukawa D, Takebayashi K, Kurihara M, Bamba S, Tani M: **The effect of preoperative synbiotic treatment to prevent surgical-site infection in hepatic resection**. *J Clin Biochem Nutr* 2020, **66**(1):67-73.

12. Gan Y, Su S, Li B, Fang C: **Efficacy of Probiotics and Prebiotics in Prevention of Infectious Complications Following Hepatic Resections: Systematic Review and Meta-Analysis**. *J Gastrointestin Liver Dis* 2019, **28**:205-211.

13. Franko J, Raman S, Krishnan N, Frankova D, Tee MC, Brahmbhatt R, Goldman CD, Weigel RJ: **Randomized Trial of Perioperative Probiotics Among Patients Undergoing Major Abdominal Operation**. *J Am Coll Surg* 2019, **229**(6):533-540.e531.

14. Yokoyama Y, Asahara T, Nomoto K, Nagino M: **Effects of Synbiotics to Prevent Postoperative Infectious Complications in Highly Invasive Abdominal Surgery**. *Ann Nutr Metab* 2017, **71 Suppl 1**:23-30.

15. Lytvyn L, Quach K, Banfield L, Johnston BC, Mertz D: **Probiotics and synbiotics for the prevention of postoperative infections following abdominal surgery: a systematic review and meta-analysis of randomized controlled trials**. *J Hosp Infect* 2016, **92**(2):130-139.

16. Yokoyama Y, Miyake T, Kokuryo T, Asahara T, Nomoto K, Nagino M: **Effect of Perioperative Synbiotic Treatment on Bacterial Translocation and Postoperative Infectious Complications after Pancreatoduodenectomy**. *Dig Surg* 2016, **33**(3):220-229.

17. van der Beek CM, Bloemen JG, van den Broek MA, Lenaerts K, Venema K, Buurman WA, Dejong CH: **Hepatic Uptake of Rectally Administered Butyrate Prevents an Increase in Systemic Butyrate Concentrations in Humans**. *J Nutr* 2015, **145**(9):2019-2024.

18. Sommacal HM, Bersch VP, Vitola SP, Osvaldt AB: **Perioperative synbiotics decrease postoperative complications in periampullary neoplasms: a randomized, double-blind clinical trial**. *Nutr Cancer* 2015, **67**(3):457-462.

19. Liu Z, Li C, Huang M, Tong C, Zhang X, Wang L, Peng H, Lan P, Zhang P, Huang N *et al*: **Positive regulatory effects of perioperative probiotic treatment on postoperative liver complications after colorectal liver metastases surgery: a double-center and double-blind randomized clinical trial**. *BMC Gastroenterol* 2015, **15**:34.

20. Zhang Y, Chen J, Wu J, Chalson H, Merigan L, Mitchell A: **Probiotic use in preventing postoperative infection in liver transplant patients**. *Hepatobiliary Surg Nutr* 2013, **2**(3):142-147.

21. Okazaki M, Matsukuma S, Suto R, Miyazaki K, Hidaka M, Matsuo M, Noshima S, Zempo N, Asahara T, Nomoto K: **Perioperative synbiotic therapy in elderly patients undergoing gastroenterological surgery: a prospective, randomized control trial**. *Nutrition* 2013, **29**(10):1224-1230.

22. Jeppsson B, Mangell P, Thorlacius H: **Use of probiotics as prophylaxis for postoperative infections**. *Nutrients* 2011, **3**(5):604-612.

23. Diepenhorst GM, van Ruler O, Besselink MG, van Santvoort HC, Wijnandts PR, Renooij W, Gouma DJ, Gooszen HG, Boermeester MA: **Influence of prophylactic probiotics and selective decontamination on bacterial translocation in patients undergoing pancreatic surgery: a randomized controlled trial**. *Shock* 2011, **35**(1):9-16.

24. Rifatbegovic Z, Mesic D, Ljuca F, Zildzic M, Avdagic M, Grbic K, Agic M, Hadziefendic B: **Effect of probiotics on liver function after surgery resection for malignancy in the liver cirrhotic**. *Med Arh* 2010, **64**(4):208-211.

25. Pitsouni E, Alexiou V, Saridakis V, Peppas G, Falagas ME: **Does the use of probiotics/synbiotics prevent postoperative infections in patients undergoing abdominal surgery? A meta-analysis of randomized controlled trials**. *Eur J Clin Pharmacol* 2009, **65**(6):561-570.

26. Gurusamy KS, Kumar Y, Davidson BR: **Methods of preventing bacterial sepsis and wound complications for liver transplantation**. *Cochrane Database Syst Rev* 2008(4):Cd006660.

27. Rayes N, Seehofer D, Theruvath T, Mogl M, Langrehr JM, Nüssler NC, Bengmark S, Neuhaus P: **Effect of enteral nutrition and synbiotics on bacterial infection rates after pylorus-preserving pancreatoduodenectomy: a randomized, double-blind trial**. *Ann Surg* 2007, **246**(1):36-41.

28. Anderson AD, McNaught CE, Jain PK, MacFie J: **Randomised clinical trial of synbiotic therapy in elective surgical patients**. *Gut* 2004, **53**(2):241-245.

29. Woodcock NP, McNaught CE, Morgan DR, Gregg KL, MacFie J: **An investigation into the effect of a probiotic on gut immune function in surgical patients**. *Clin Nutr* 2004, **23**(5):1069-1073.

30. McNaught CE, Woodcock NP, MacFie J, Mitchell CJ: **A prospective randomised study of the probiotic Lactobacillus plantarum 299V on indices of gut barrier function in elective surgical patients**. *Gut* 2002, **51**(6):827-831.
