# Supplementary material for: The effect of perioperative probiotics and synbiotics on postoperative infections in patients undergoing major liver surgery: a meta-analysis of randomized controlled trials
Source: PeerJ. 2025 Feb 17;13:e18874. doi: 10.7717/peerj.18874 (PMC11841616; doi:10.7717/peerj.18874)
Supplement: Supplemental Information 4 [file peerj-13-18874-s004.docx]

**Supplementary Material 4:** Heatmap, publication bias assessment by funnel plot and Egger’s test, sensitivity analyses, subgroup analyses.


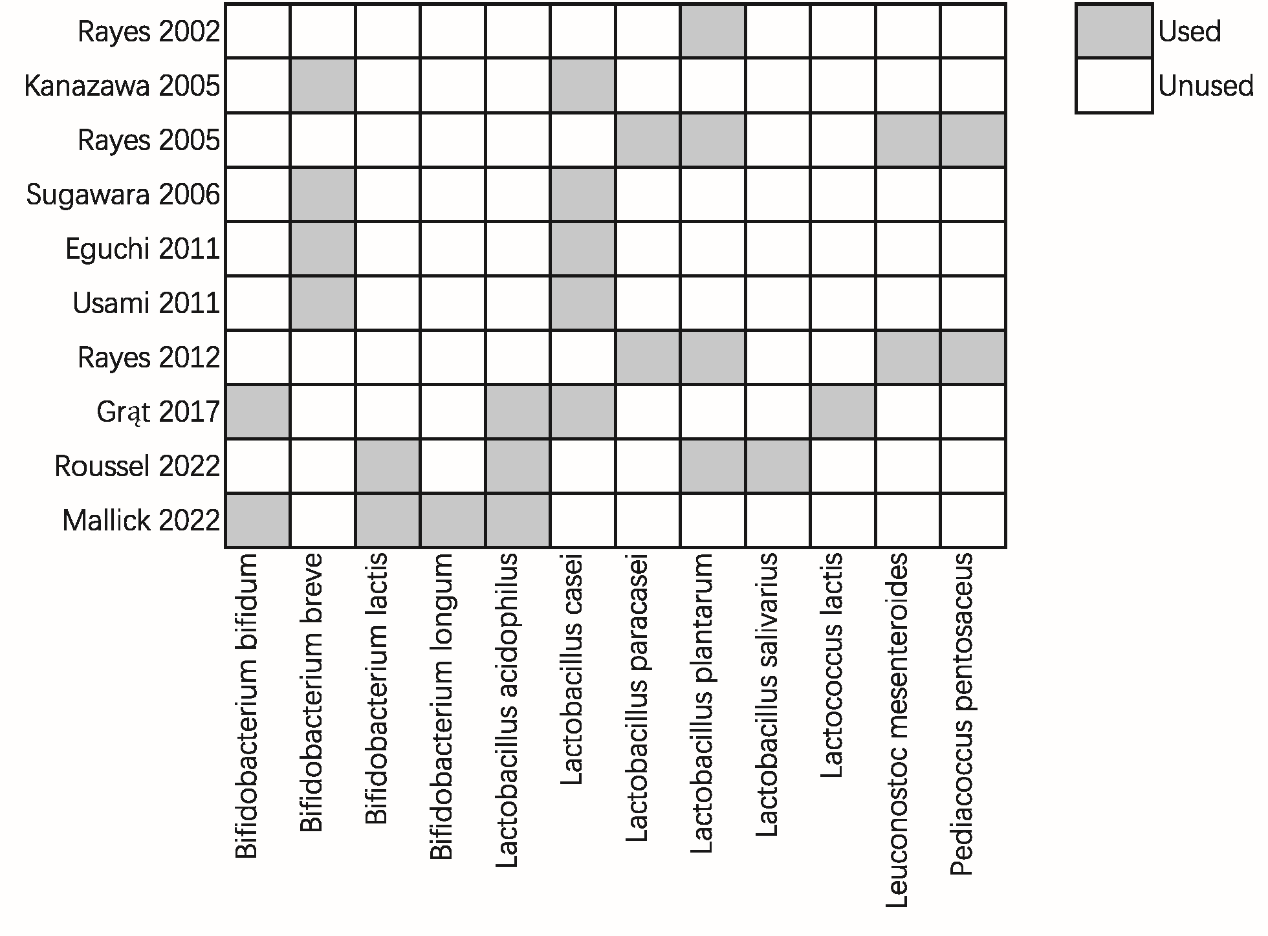
 Figure 1: Heatmap shows the different probiotic species used in different studies


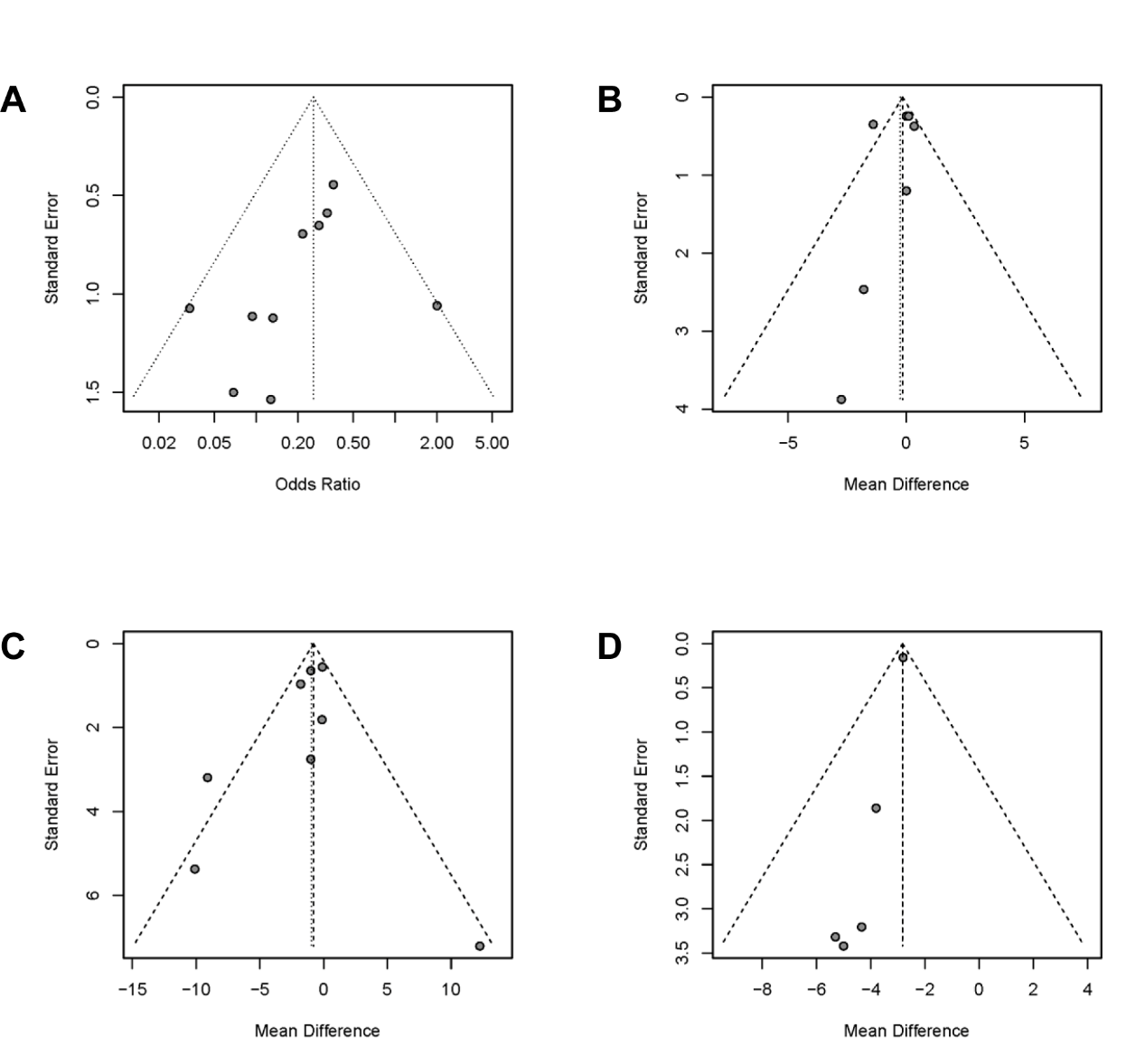


Figure 2: Funnel plot for (A) postoperative infections, Egger’s test P=0.1781, (B) length of ICU stay, Egger’s test P=0.5458, (C) length of hospital stay, Egger’s test P= 0.4325, (D) length of antibiotic therapy, Egger’s test P=0.0020


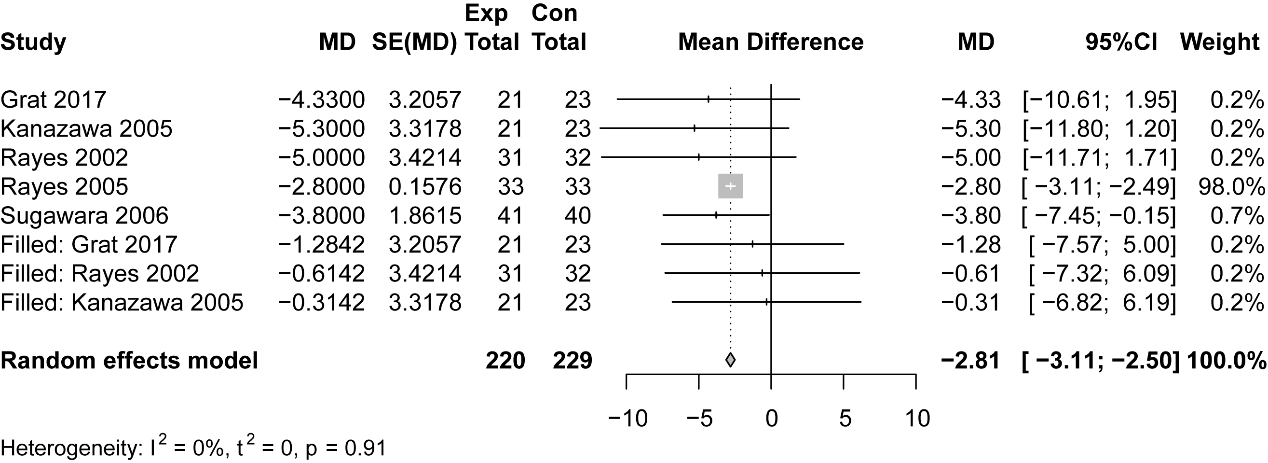


Figure 3: Forest plot for length of antibiotic therapy after trim-and-fill method


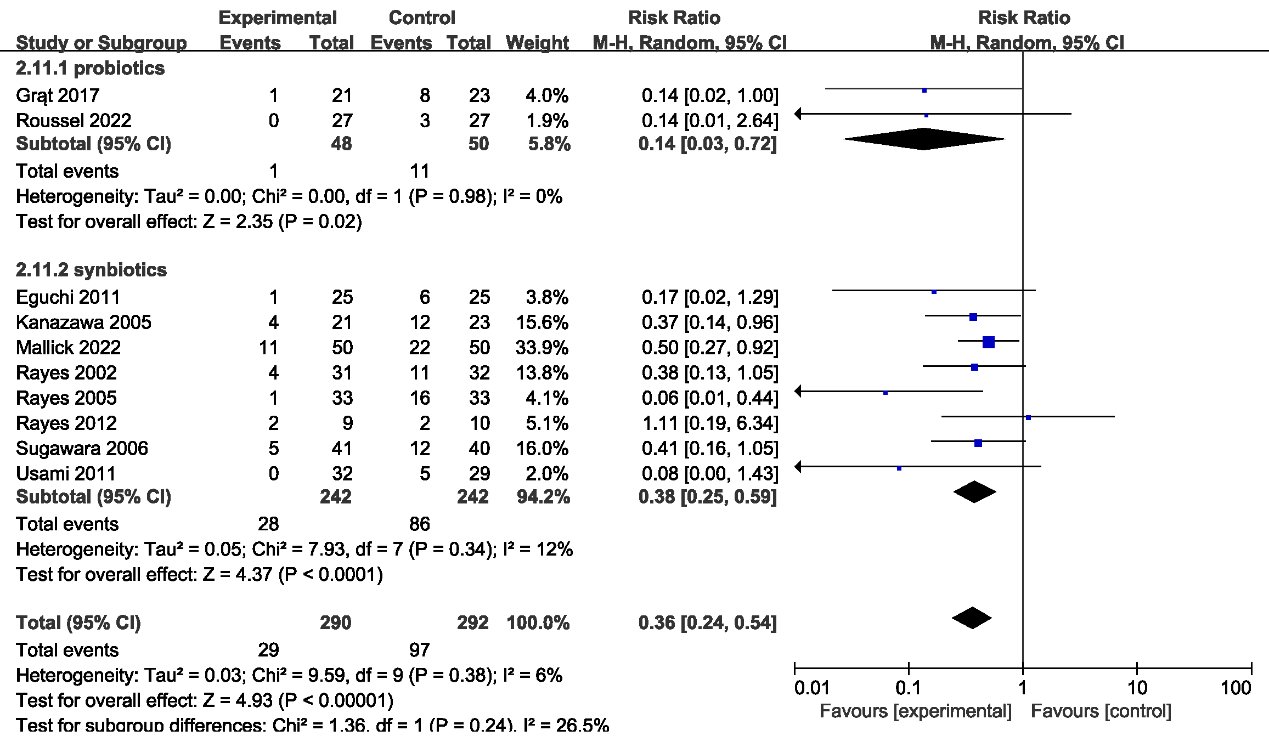


Figure 4: Forest plot showing the subgroup analysis of postoperative infections, probiotics versus synbiotics


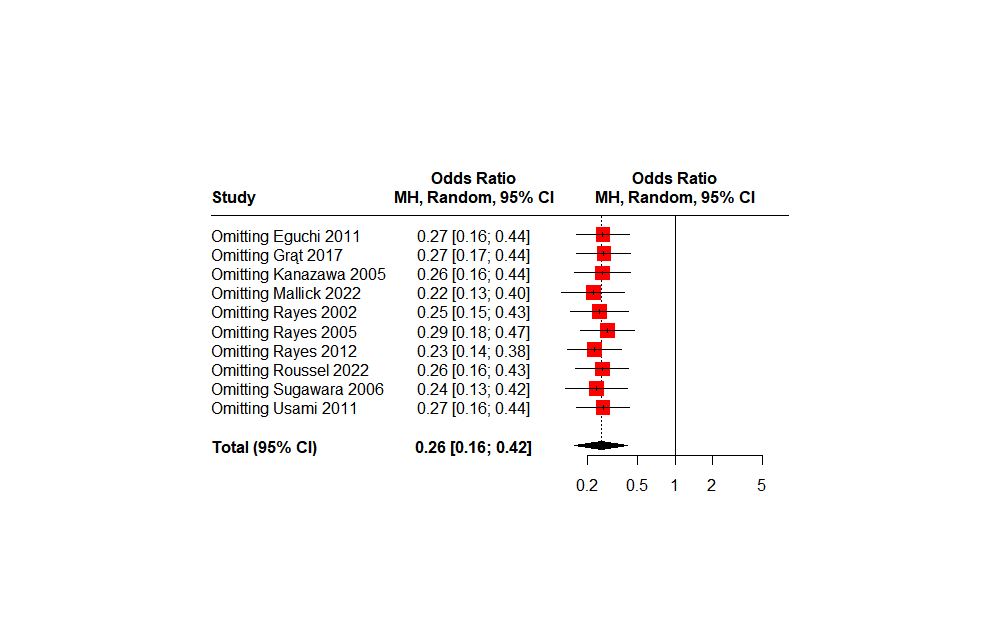


Figure 5: Sensitivity analysis for postoperative infections


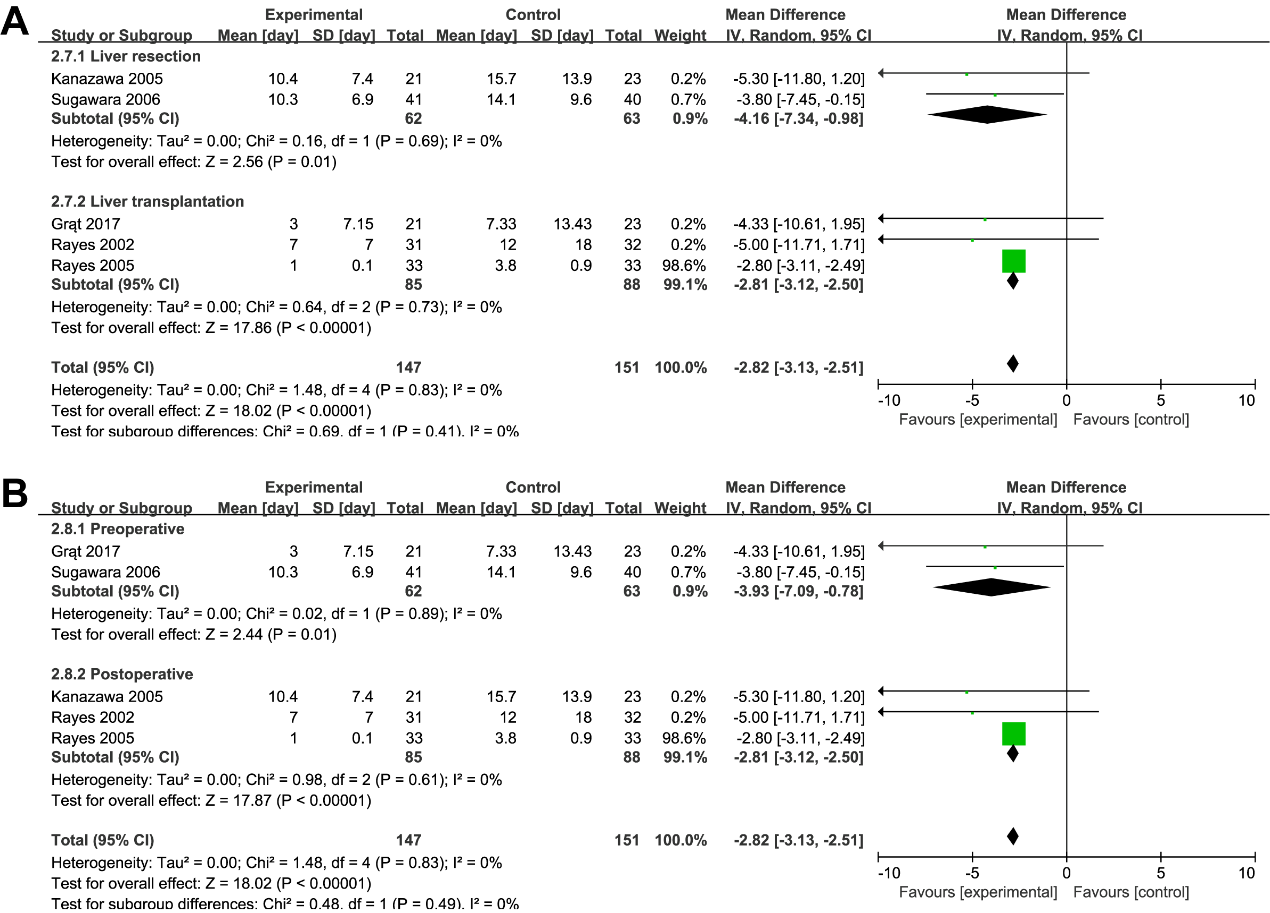


Figure 6: Forest plot showing the subgroup analysis of length of antibiotic therapy, (A)liver resection versus liver transplantation; (B) preoperative versus postoperative


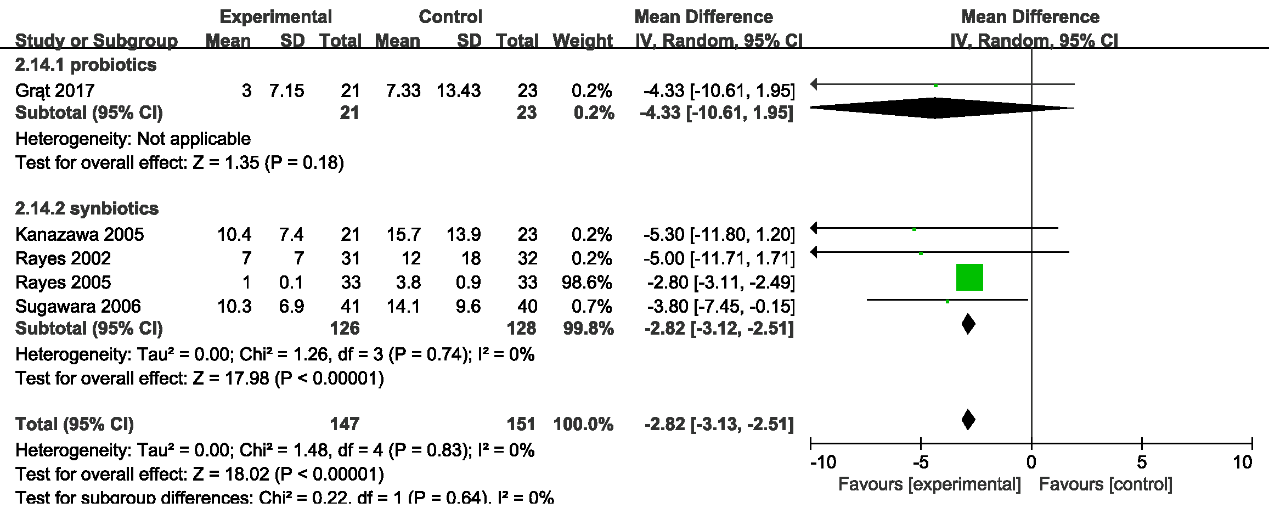


Figure 7: Forest plot showing the subgroup analysis of length of antibiotic therapy, probiotics versus synbiotics


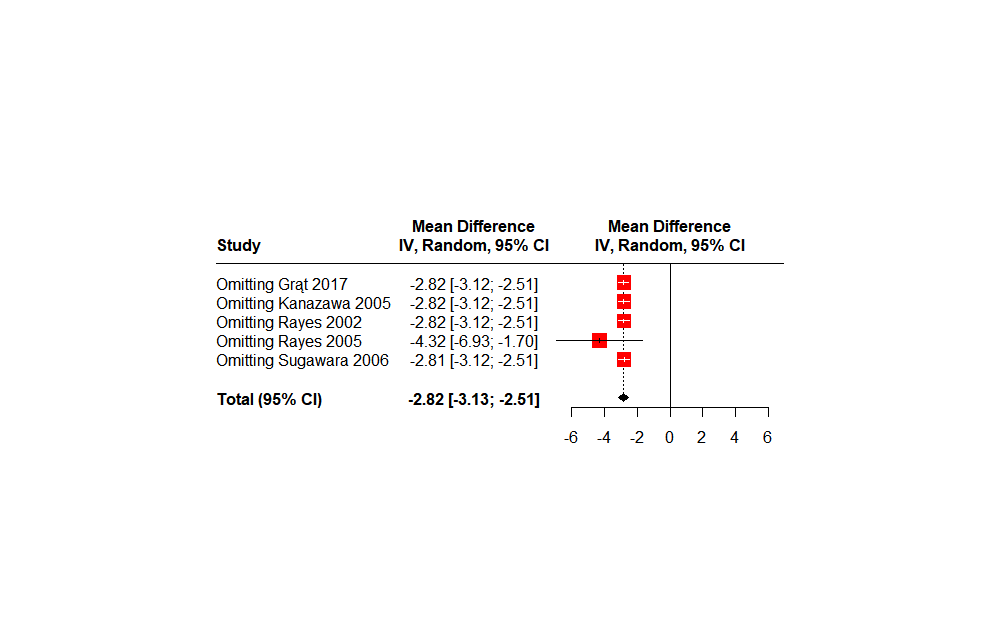


Figure 8: Sensitivity analysis for length of antibiotic therapy


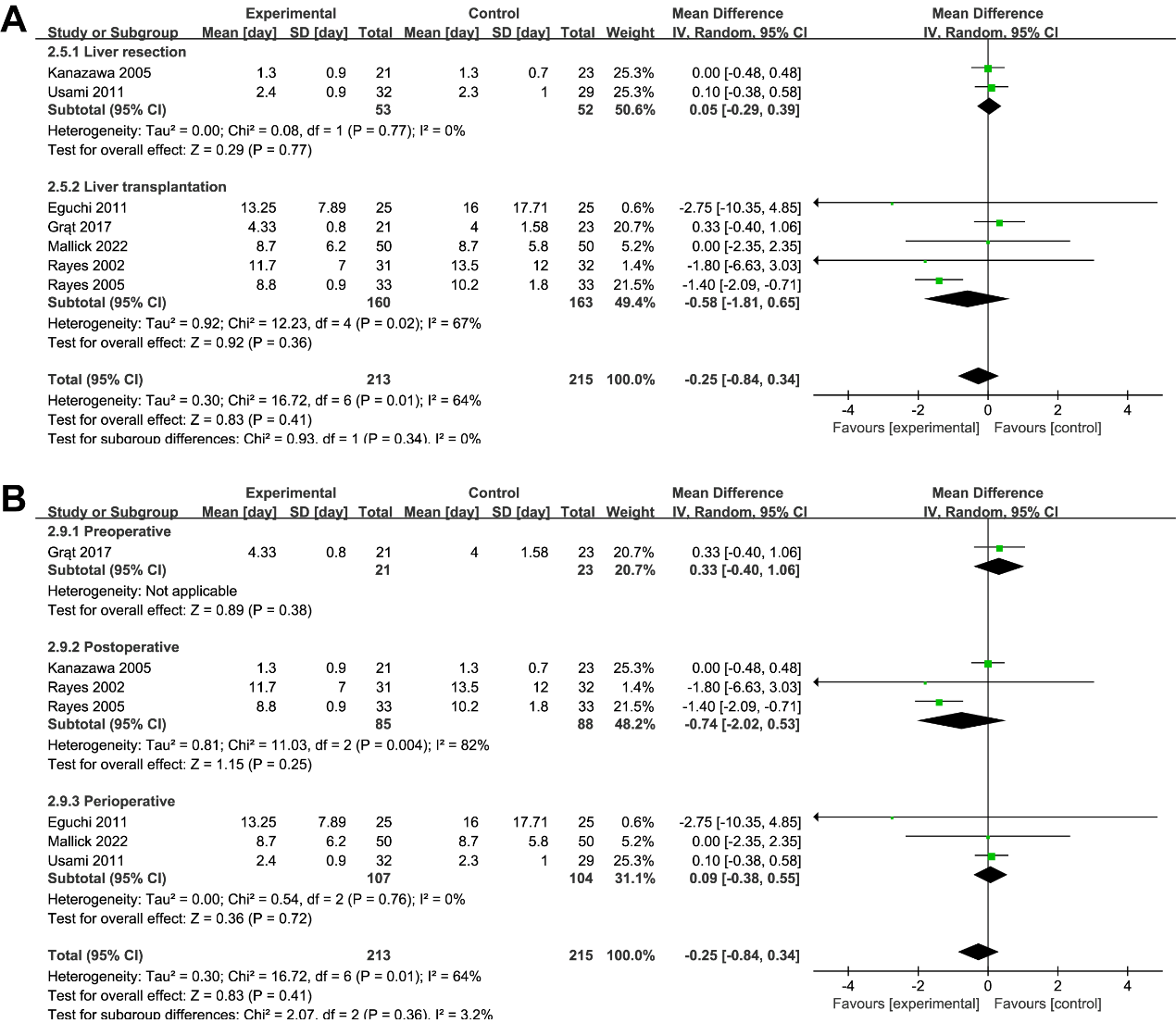


Figure 9: Forest plot showing the subgroup analysis of length of ICU stay, (A)liver resection versus liver transplantation; (B) preoperative versus postoperative


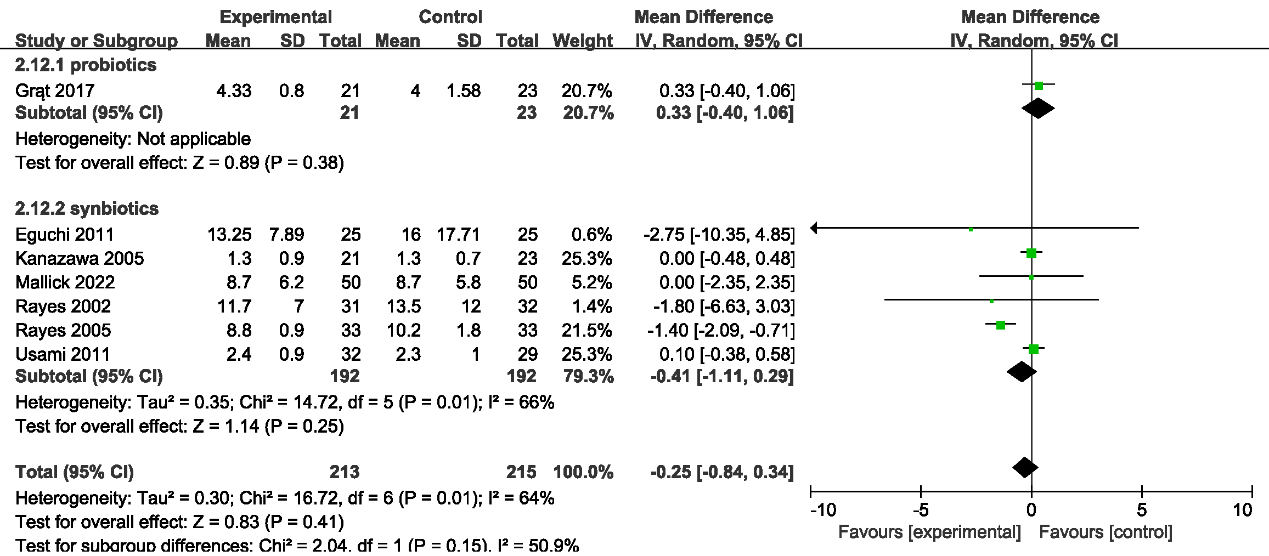


Figure 10: Forest plot showing the subgroup analysis of length of ICU stay, probiotics versus synbiotics


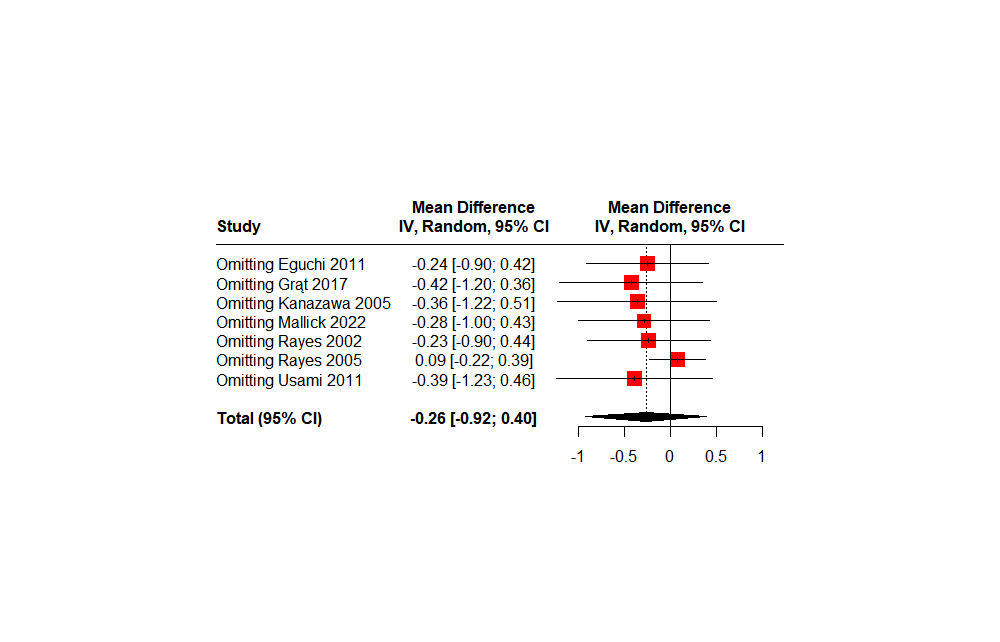


Figure 11: Sensitivity analysis for length of ICU stay


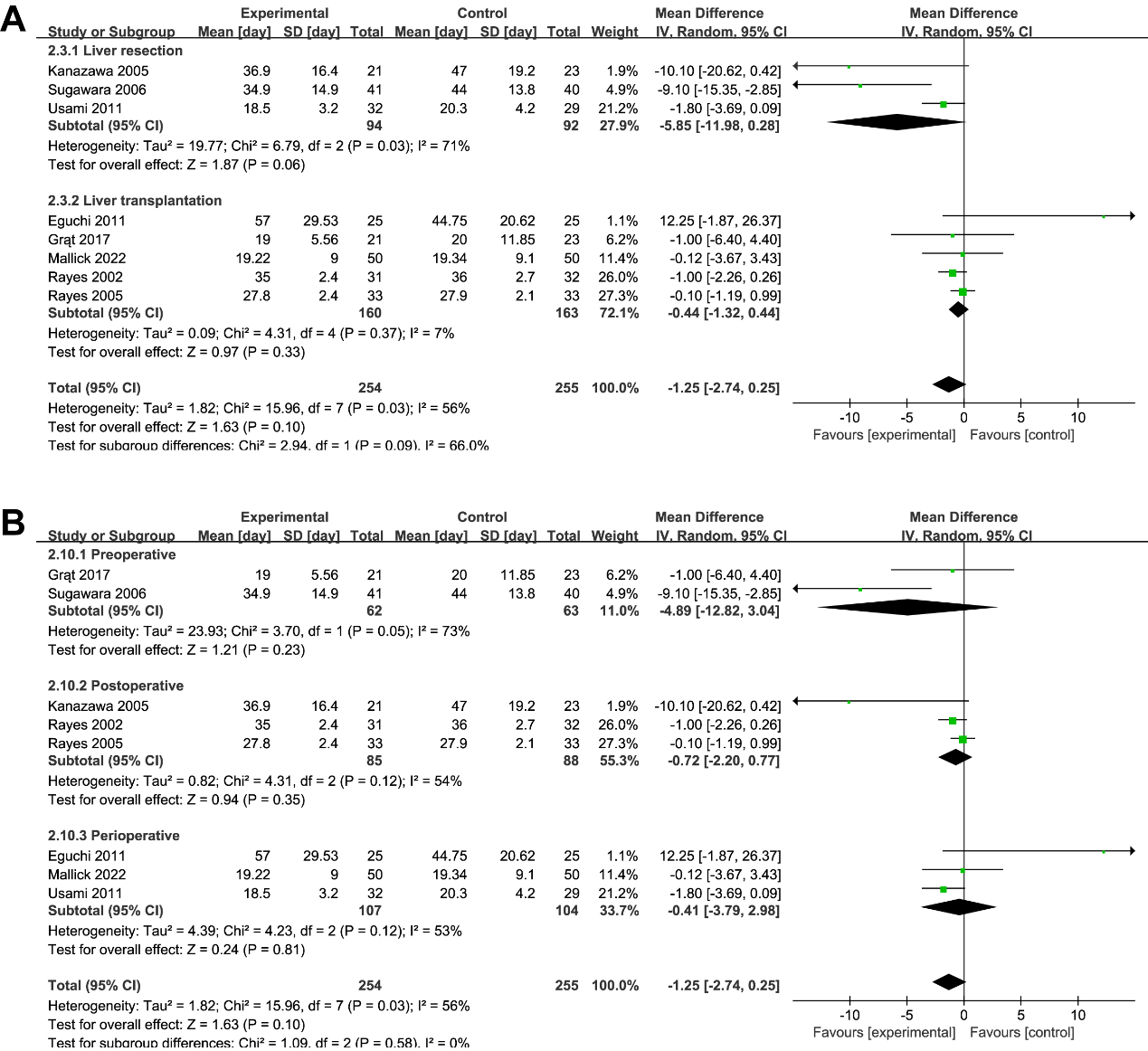


Figure 12: Forest plot showing the subgroup analysis of length of hospital stay, (A)liver resection versus liver transplantation; (B) preoperative versus postoperative


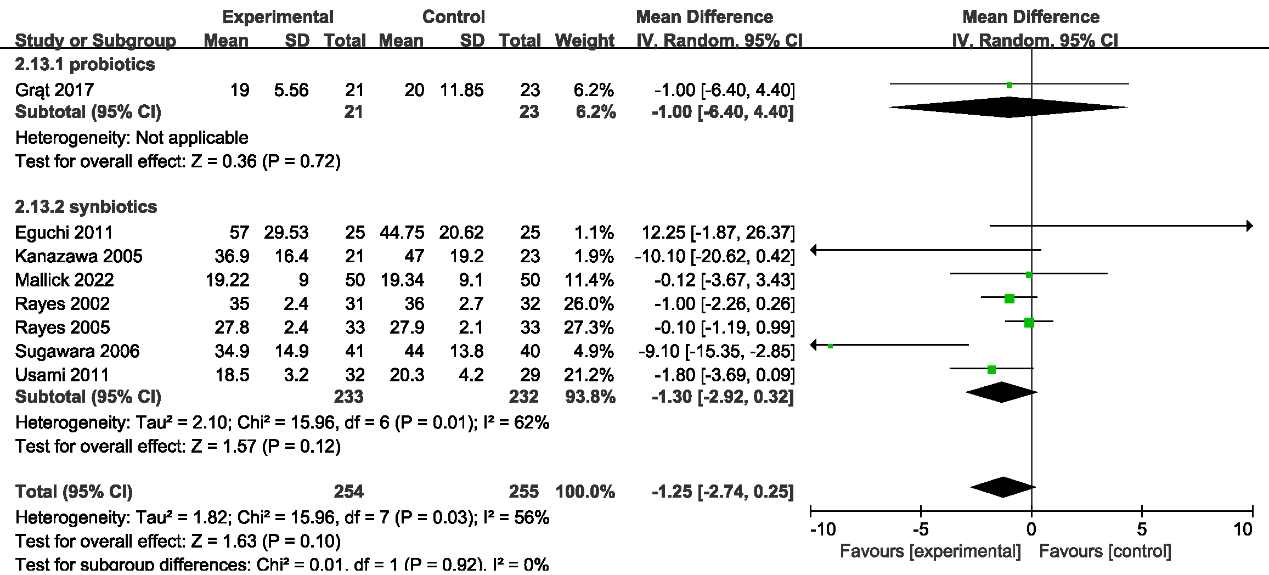


Figure 13: Forest plot showing the subgroup analysis of length of hospital stay, probiotics versus synbiotics


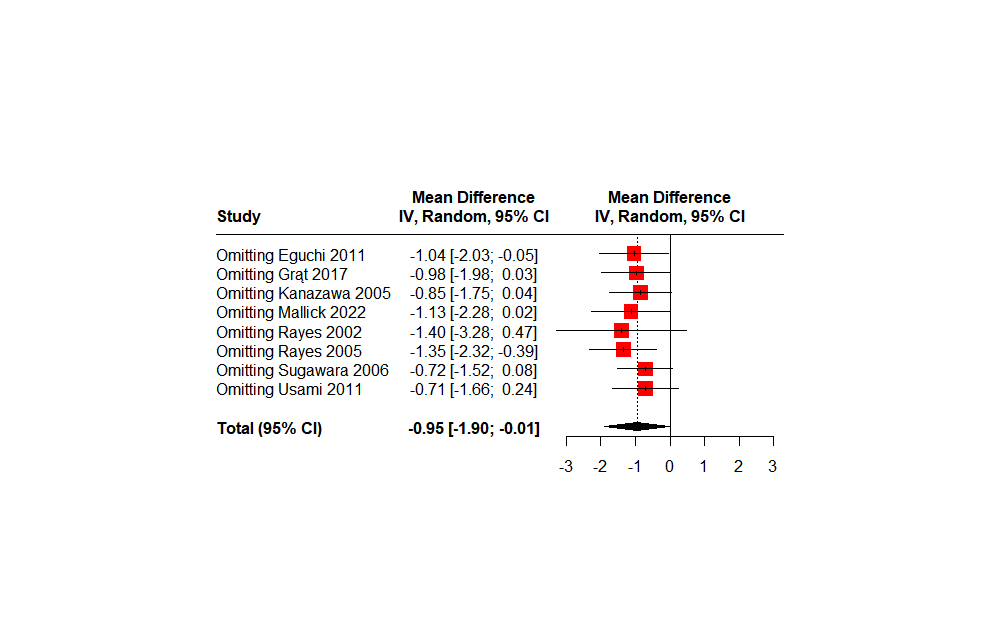


Figure 14: Sensitivity analysis for length of hospital stay


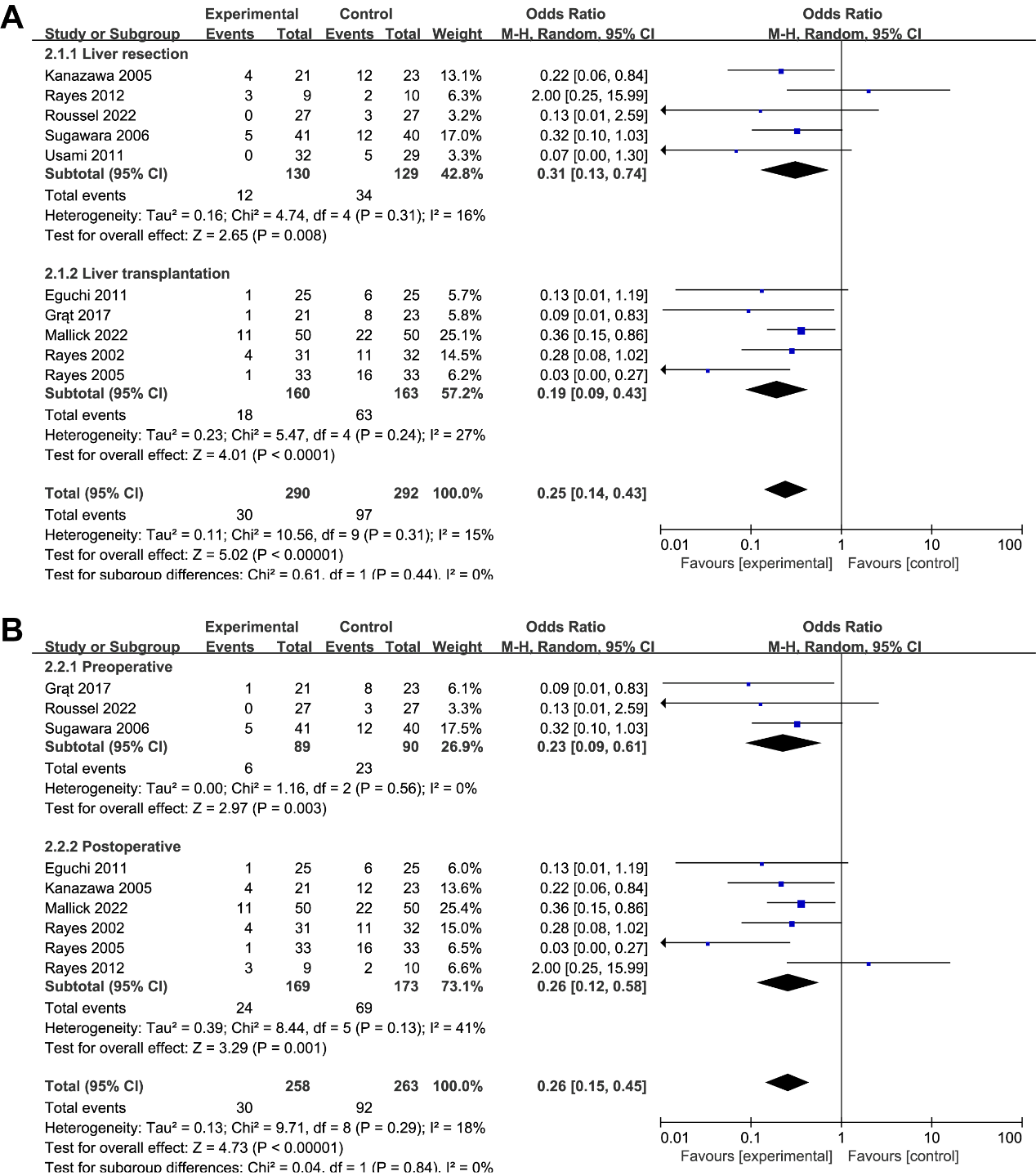
Figure 11: Subgroup analysis for postoperative infections, (A) liver resection versus liver transplantation, (B) preoperative versus postoperative


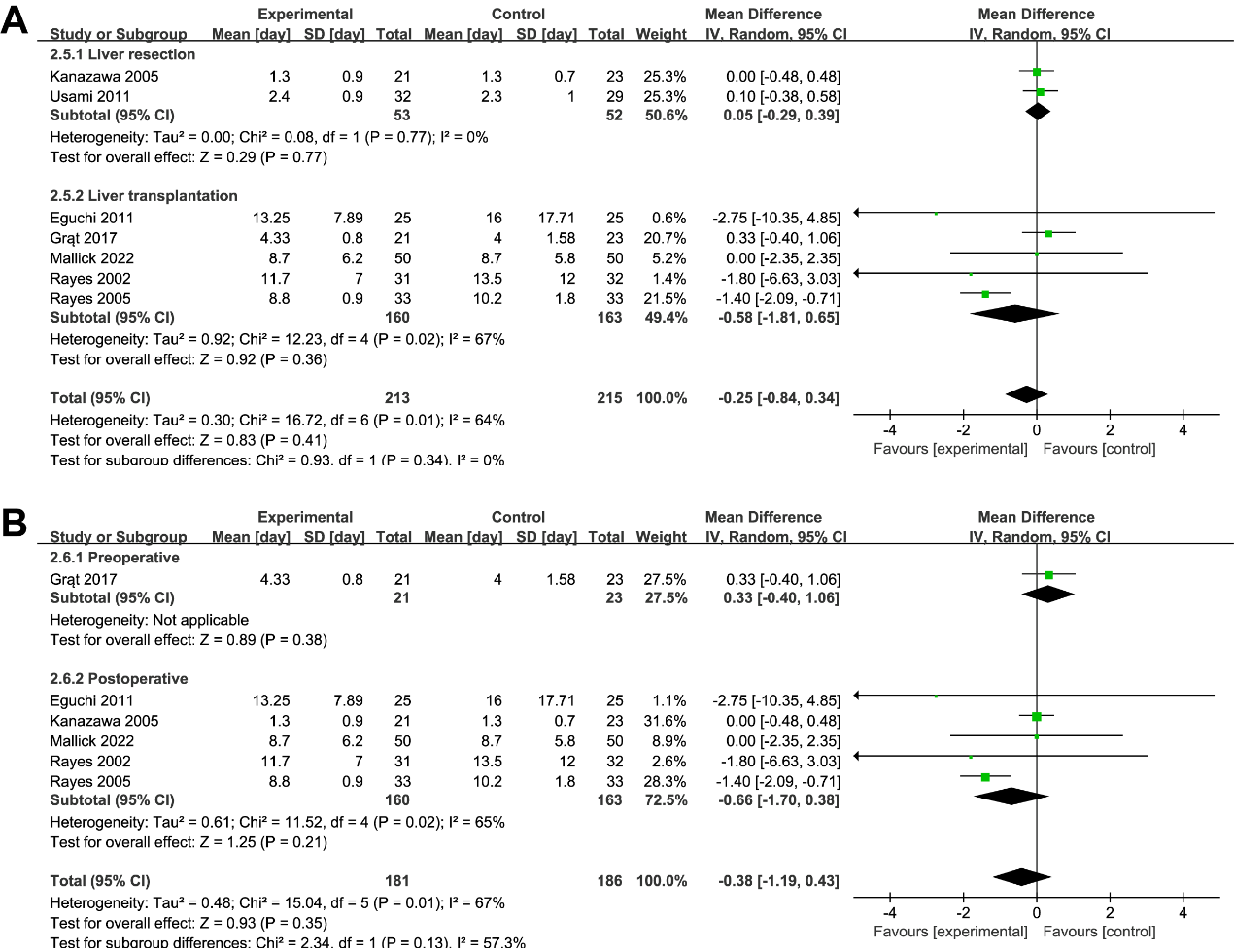


Figure 12: Subgroup analysis for length of ICU stay, (A) liver resection versus liver transplantation, (B) preoperative versus postoperative


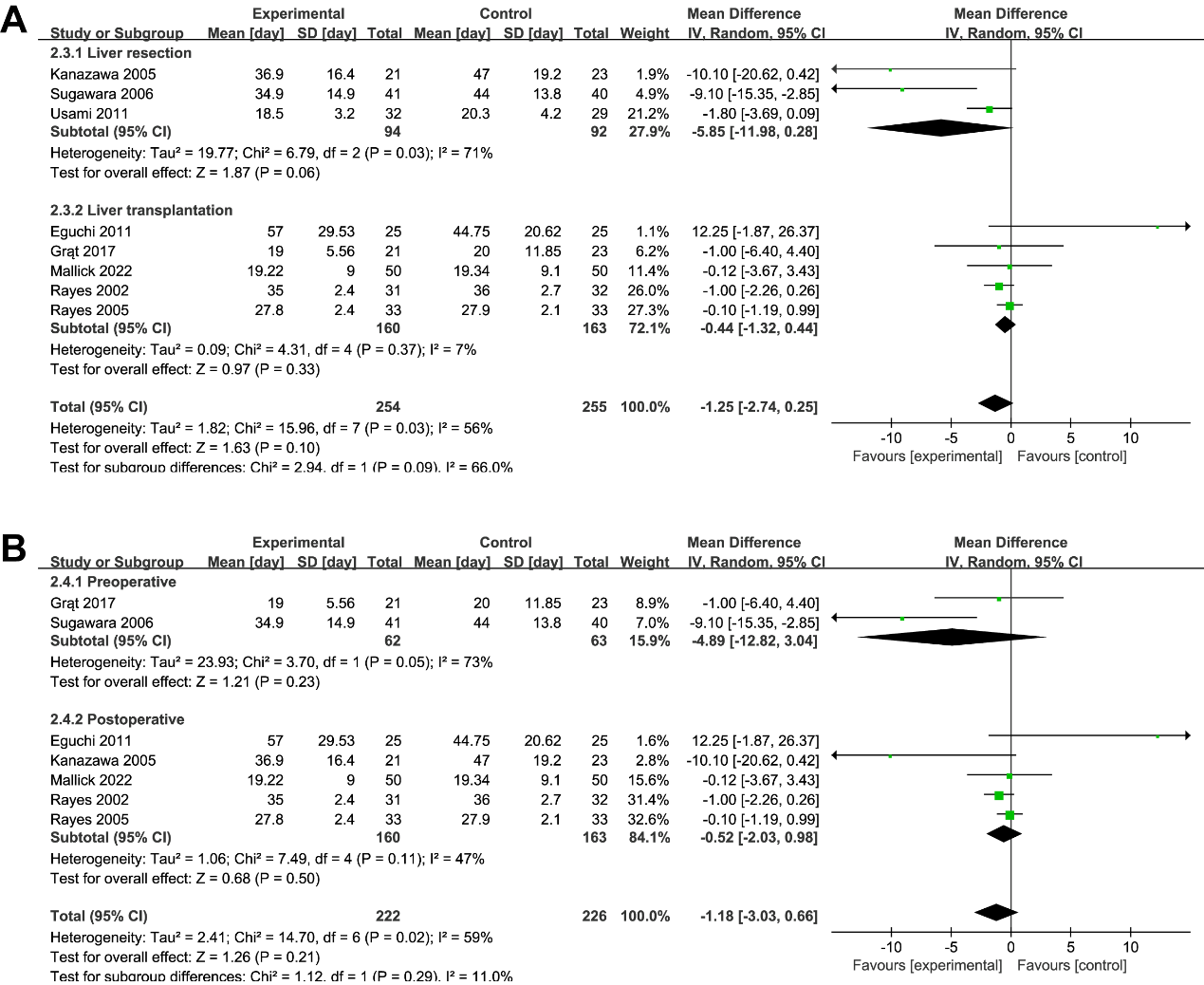


Figure 13: Subgroup analysis for length of hospital stay, (A) liver resection versus liver transplantation, (B) preoperative versus postoperative


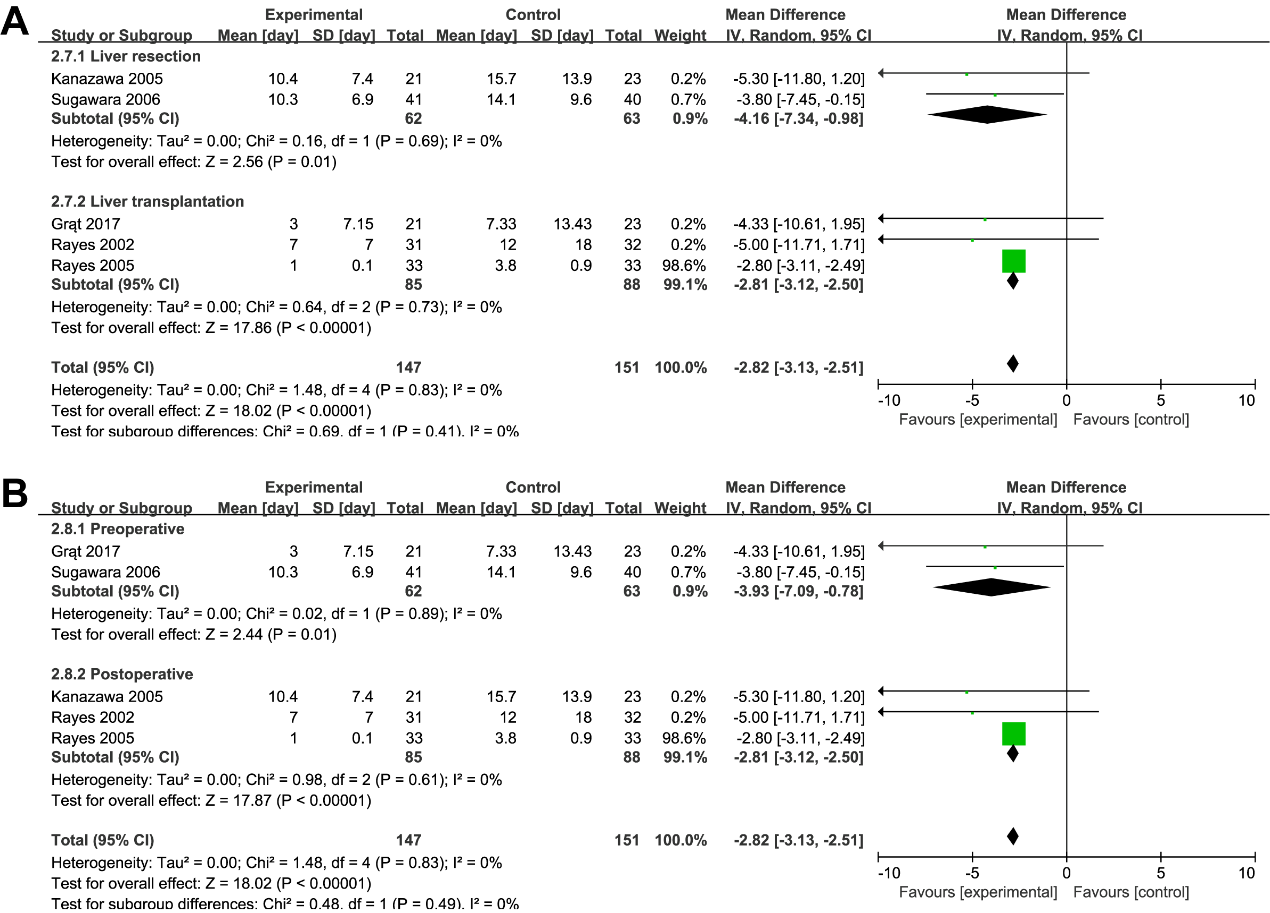


Figure 14: Subgroup analysis for length of antibiotic therapy, (A) liver resection versus liver transplantation, (B) preoperative versus postoperative
