# Supplementary material for: The effect of perioperative probiotics and synbiotics on postoperative infections in patients undergoing major liver surgery: a meta-analysis of randomized controlled trials
Source: PeerJ. 2025 Feb 17;13:e18874. doi: 10.7717/peerj.18874 (PMC11841616; doi:10.7717/peerj.18874)
Supplement: Supplemental Information 5 [file peerj-13-18874-s005.pdf]

The effect of perioperative probiotics and synbiotics on postoperative infections in patients undergoing major liver surgery

A meta-analysis of 10 randomized controlled trials

POPULATION

Adult patients undergoing major liver surgeries (including liver resections and transplantations)

INTERVENTION

Enteral probiotics/synbiotics

Enteral placebo

OUTCOMES

Postoperative infections
10 RCTs, 582 participants

Duration of antibiotic therapy
5 RCTs, 298 participants

Length of ICU stay
7 RCTs, 428 participants

Length of hospital stay
8 RCTs, 509 participants

FINDINGS

Outcomes	Risk Ratio (95% CI)
**Postoperative infections**	0.36 (0.24-0.54)
**Surgery types**	
Liver resection	0.39 (0.21-0.72)
Liver transplantation	0.28 (0.13-0.59)
**Timing**	
Preoperative	0.31 (0.14-0.71)
Postoperative	0.27 (0.11-0.67)
Perioperative	0.44 (0.21-0.95)
**Intervention**	
Probiotics	0.14 (0.03-0.72)
Synbiotics	0.38 (0.25-0.59)

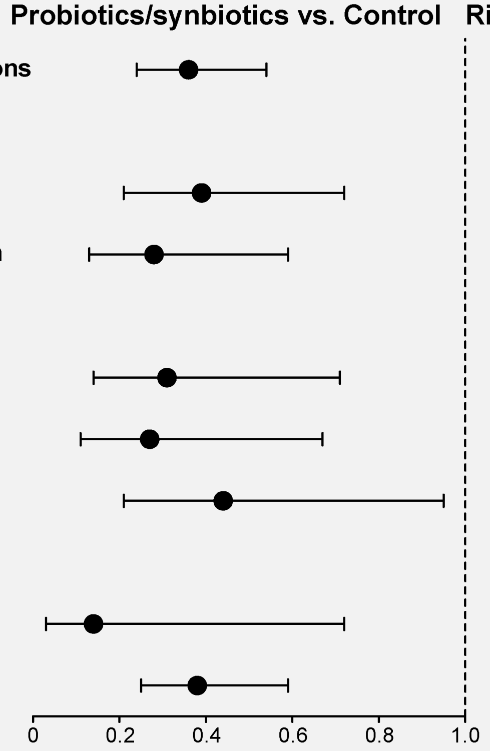

Duration of antibiotic therapy MD -2.82 (-3.13, -2.51)

Length of ICU stay MD -0.25 (-0.84, 0.34)

Length of hospital stay MD -1.25 (-2.74, 0.25)
